# Hepatotoxicity of pyrrolizidine alkaloids in rats in relation to human exposure

**DOI:** 10.1007/s00204-020-02850-y

**Published:** 2020-07-16

**Authors:** Hermann M. Bolt

**Affiliations:** Leibniz Research Centre for Working Environment and Human Factors, Ardeystr. 67, 44139 Dortmund, Germany

Recently, Ebmeyer and colleagues published a 28-day feeding study with six pyrrolizidine alkaloids in rats (Ebmeyer et al. 2020). Pyrrolizidine alkaloids occur in honey, tea and herbal spices, which represent the main sources of human exposure (Bodi et al. 2014; EFSA 2017; Mulder et al. 2018). They consist of a common basic structure, the 1-hydroxymethylpyrrolizidine, esterified with one or two aliphatic mono- or dicarboxylic acids (Stegelmeier et al. 1999; Wiedenfeld 2011). Uptake of high amounts of contaminated food may cause a human health risk (BfR 2018, 2019). Recently, it has been shown that single acute doses of senecionine to mice induced cytochrome P450-dependent destruction of sinusoidal endothelial cells (Hessel-Pras et al. 2020). However, relatively little is known about subchronic and chronic toxicity with doses relevant for human exposure. Therefore, Ebmeyer and colleagues used not acutely toxic doses of 0.1 to 3.3 mg/kg body weight for a 28-day study in rats and performed genome-wide expression analyses of liver tissue. These doses are of human relevance, because consumption of contaminated tea may result in doses of ~ 8 µg/kg in adults, while pyrrolizidine exposure of up to 3 mg/kg body weight was reported for infants, who consumed contaminated food. Liver enzymes were not increased in the exposed rats. However, at the highest tested doses, gene array analyses showed clear expression changes (Ebmeyer et al. 2020). A set of 36 commonly regulated genes was observed for the high-dose groups of four structurally different pyrrolizidine alkaloids. Among them, genes associated with DNA damage response and cell cycle regulation were enriched.

Hepatotoxicity represents a major focus in current toxicological research (Jansen et al. 2017; Ghallab et al. 2019a). Current studies with intravital imaging (Ghallab et al. 2019b; Reif et al. 2017) and modeling (Vartak et al. 2016) give new insight into the complex interplay between the individual liver cell types during damage induction (Hoehme et al. 2010; Ghallab et al. 2016). Interspecies extrapolation remains a major challenge when toxicity studies are based on animal experiments (Thiel et al. 2015; Schenk et al. 2017); differences between the in vitro and in vivo situations represent not yet sufficiently solved challenges when human cell systems are used (Grinberg et al. 2014, 2018; Godoy et al. 2013, 2016).

Often risk evaluation is hampered by a lack of carefully performed subchronic or chronic animal studies with human relevant doses. Therefore, the present study of Ebmeyer et al. represents an important milestone in the current research on the hepatotoxicity of pyrrolizidine alkaloids.
